# Diagnostics of pediatric supratentorial RELA ependymomas: integration of information from histopathology, genetics, DNA methylation and imaging

**DOI:** 10.1111/bpa.12664

**Published:** 2018-11-28

**Authors:** Mélanie Pagès, Kristian W. Pajtler, Stéphanie Puget, David Castel, Nathalie Boddaert, Arnault Tauziède‐Espariat, Stéphanie Picot, Marie‐Anne Debily, Marcel Kool, David Capper, Christian Sainte‐Rose, Fabrice Chrétien, Stefan M. Pfister, Torsten Pietsch, Jacques Grill, Pascale Varlet, Felipe Andreiuolo

**Affiliations:** ^1^ Department of Neuropathology Sainte‐Anne Hospital Paris France; ^2^ Paris V Descartes University, Paris Cité Sorbonne Paris France; ^3^ Institut National de la Santé et de la recherche Médicale, INSERM Unit 1000 « Neuroimaging & Psychiatrie », Université Paris Sud Orsay France; ^4^ Hopp Children’s Cancer Centre at the NCT (KiTZ) Heidelberg Germany; ^5^ Division of Pediatric Neurooncology German Cancer Research Centre (DKFZ) and German Cancer Consortium (DKTK) Heidelberg Germany; ^6^ Department of Hematology and Oncology Heidelberg University Hospital Heidelberg Germany; ^7^ Department of Pediatric Neurosurgery Necker Enfants Malades Hospital Paris France; ^8^ UMR8203 « Vectorologie et Thérapeutiques Anticancéreuses » Centre National de la Recherche Scientifique; ^9^ Département de Cancérologie de l’Enfant et de l’Adolescent Gustave Roussy, Univ. Paris‐Sud, Université Paris‐Saclay Villejuif France; ^10^ Radiology Department Necker Enfants Malades Hospital AP‐HP Paris France; ^11^ Institut National de la Santé et de la recherche Médicale, INSERM UMR 1163, Institut Imagine, and INSERM U1000 Paris France; ^12^ Université d’Evry‐Val d’Essonne Evry France; ^13^ Department of Neuropathology Charité – Universitätsmedizin Berlin, corporate member of Freie Universität Berlin, Humboldt‐Universität zu Berlin, and Berlin Institute of Health Berlin Germany; ^14^ German Cancer Consortium (DKTK), Partner Site Berlin, German Cancer Research Centre (DKFZ) Heidelberg Germany; ^15^ Infection & Epidemiology Department Human Histopathology and Animal Models Unit, Institut Pasteur Paris France; ^16^ Institute of Neuropathology University of Bonn Medical Centre Bonn Germany

**Keywords:** C11orf95‐RELA, FISH, HGNET, subependymoma, Supratentorial Ependymoma, YAP1

## Abstract

Ependymoma with *RELA* fusion has been defined as a novel entity of the revised World Health Organization 2016 classification of tumors of the central nervous system (CNS), characterized by fusion transcripts of the *RELA* gene and consequent pathological activation of the NFkB pathway. These tumors represent the majority of supratentorial ependymomas in children. The validation of diagnostic tools to identify this clinically relevant ependymoma entity is essential. Here, we have used interphase fluorescent *in situ* hybridization (FISH) for *C11orf95 *and *RELA*, immunohistochemistry (IHC) for p65‐RelA and the recently developed DNA methylation‐based classification besides conventional histopathology, and compared the precision of the methods in 40 supratentorial pediatric brain tumors diagnosed as ependymomas in the past years. Reverse transcription PCR (RT‐PCR) and RNA sequencing were performed to explore discordant cases. Furthermore, we integrated imaging and clinical features as additional layers of information. The concordance between nuclear RelA expression by IHC and *RELA* FISH was 100%. Concordance between IHC and DNA methylation profiling, and between FISH and DNA methylation profiling was also high (96.4% and 95.2%, respectively). Thirty‐four out of 40 (85%) cases were confirmed by integrated diagnoses as ependymal tumors, including 22 *RELA*‐fused ependymomas (71% of ependymal tumors), two *YAP1‐*fused ependymomas (6%), six non‐*RELA*/non‐*YAP1* ependymomas (18%) and four ependymal/subependymal mixed tumors (12%). Ependymal/subependymal mixed tumors had an excellent clinical outcome despite the presence of histopathological signs of malignancy, suggesting that these tumors should not be diagnosed as classic ependymomas. DNA methylation profiling helped in the differential diagnosis of *RELA‐*fused ependymomas. IHC and FISH, which are available in the majority of pathology laboratories, are valuable tools to identify *RELA*‐fused ependymomas.

## Introduction

Ependymoma is a rare tumor of the central nervous system (CNS) occurring all along the neuroaxis. Almost 90% of pediatric ependymomas are intracranial, of which around one‐third arise in the supratentorial compartment, with a mean age of around 8 years at diagnosis 12. Ependymomas in children have shown to have a poor prognosis with 65% progression‐free survival at 5 years 11. Although histopathological grading has been found to be associated with outcome in some recent studies 1, 10, 11, the criteria for grading according to the World Health Organization (WHO) classification are not well defined. The inter‐observer variance in grading is high and such grading has failed to correlate with prognosis in several cohorts of intracranial ependymomas 4, 7. Recently, a specific translocation was identified in around 2/3 of supratentorial ependymoma in children, involving the genes *RELA *(*v‐rel avian reticuloendotheliosis viral oncogene homolog A*) and *C11orf95, *resulting in an oncogenic fusion gene, leading to nuclear translocation of p65‐RelA protein and pathological activation of NFκB signaling. In a smaller proportion of cases, other genes can be involved in fusion events such as *YAP1* 17, 19. Ependymomas with *RELA* fusion were defined as a novel entity in the 2016 revision of the WHO classification of tumors of the CNS 9. Among supratentorial ependymomas, DNA methylation profiling identified three molecular subgroups, namely “EPN, RELA” (72%) including all cases harboring *RELA* fusions, “EPN, YAP” (11%) including cases harboring *YAP* fusions and “SUBEPN, ST” (17%) for which no gene fusions or other driver genes have been identified yet 17. However, this cohort included both pediatric and adult patients and the study did not correlate the epigenetic pattern with histopathological and molecular genetic features, while histopathology remains the standard for ependymoma diagnosis according the WHO classification 2016, except for the *RELA*‐fused ependymomas 9. Otherwise, although alternative fusions have been described 17, 19, ependymomas outside RELA, YAP or subependymoma subgroups were not referred to in the classification proposed in this previous work 17. Interestingly, the “EPN, RELA” subgroup showed a significantly worse clinical outcome compared to “EPN, YAP” and “SUBEPN, ST” in this retrospective case collection.

Based on these recent advances to identify clinically and molecularly distinct subgroups of ependymomas, an international meeting generated a series of consensus statements and recommendations which comment on the prognostic evaluation and treatment decisions of children with intracranial ependymoma 18. In this context, it is of paramount importance to validate additional tools to identify different ependymoma subtypes that are applicable and available for routine diagnosis and clinical practice, in particular for the diagnostics of *RELA* fusion‐positive ependymomas.

The aim of the present study was therefore to compare the precision of fluorescent *in situ* hybridization (FISH), immunohistochemistry (IHC) for nuclear RelA accumulation and DNA methylation‐based classification, besides conventional histopathology in the diagnostics of *RELA* ependymomas in a retrospective series of 40 pediatric brain tumors diagnosed as supratentorial ependymomas by histopathological assessment in the past. Furthermore, we integrated imaging and clinical data as additional information.

## Materials and Methods

### Patients and tumor samples

We studied 40 pediatric patients with tumors initially diagnosed as supratentorial ependymomas by histopathological assessment between 1993 and 2014, retrieved from the archives of Sainte‐Anne and Necker‐Enfants‐Malades Hospitals in Paris. Patients were operated at Necker and treated at Gustave Roussy. The use of clinical data and biologic material was reviewed in conformity with the institutional internal review boards. All patients were treated by maximal safe surgical excision, followed by radiotherapy in patients older than three years and adjuvant chemotherapy in patients younger than three years and/or incomplete surgical excision. All cases included had the neuropathological diagnosis of supratentorial ependymoma in the initial pathological report. Cases diagnosed as subependymomas (WHO grade I) were not included. Histopathological re‐review by senior pathologists (PV and FA) according to the revised WHO 2016 classification was performed subsequently. The cases were also subjected to local radiological (NB) review (Magnetic resonance imaging exams and if available computed tomography preoperative scans). The Kaplan–Meier analyses were performed for survival data using the log‐rank test. The level of significance was *P* < 0.05. Analyses were performed using IBM SPPSS statistics software (v20).

### Immunohistochemistry

Immunohistochemistry for p65‐RelA, YAP1, Tenascin C, H3‐K27M, H3‐K27me3 and BCOR were performed as described previously 1, 16. p65‐RelA IHC was independently analyzed by two senior pathologists (FA and PV) blinded for the FISH and DNA methylation results. For details, see supporting information.

### FISH

FISH assessment was performed on interphase nuclei on 4‐µm thick FFPE slides, as previously described 15. *RELA* break‐apart probes were derived from BAC clones (Empire Genomics, Buffalo, NY, USA), covering 3′*RELA* and 5′*RELA *regions on 11q13.1 (RP11‐58D3, labeled with 5‐TAMRA and RP11‐436C17/RP11‐1104L6 labeled with 5‐fluorescein‐deoxyuridine triphosphate). Metaphase FISH was performed to verify correct mapping of the clones. *C11orf95* FISH was performed using a break‐apart custom SureFISH probe and hybridized according to the manufacturer’s recommendations (Agilent Technologies, Santa Clara, CA, USA). *MN1* and *YAP1* rearrangement suspected by methylation analysis was controlled using a *MN1* break‐apart probe and a *YAP1* break‐apart probe (Empire Genomics, Buffalo, NY, USA). Signals were scored in at least 100 nonoverlapping interphase nuclei. A case was considered positive when the scored nuclei displayed a break‐apart signal in at least 20% of the counted nuclei. Gain of 1q was also assessed, using the Zyto*Light*® SPEC 1p36/1p25 Dual Color Probe kit (ZytoVision, Bremerhaven, Germany) according to the manufacturers’ instructions. Assays were considered non‐informative if the FISH signals were either lacking or too weak to be interpreted. Results were recorded using a DM6000 imaging fluorescence microscope (Leica Biosystems, Nanterre, France) fitted with appropriate filters, a CCD camera and digital imaging software (CytoVision, v7.4).

### DNA extraction and DNA methylation analysis

DNA was extracted, converted by bisulfite treatment and used for methylation‐based classification as described 3. For details, see supporting information.

### RNA sequencing

RNA sequencing was performed on three primary tumor samples. Total RNA was extracted from frozen specimen using Allprep® DNA/RNA Extraction kit from Qiagen (Hilden, Germany). Libraries were prepared from 1 µg of total RNA using the TruSeq Stranded mRNA Sample preparation kit (Illumina, San Diego, CA, USA) following the supplier recommendations. Paired‐end sequencing was carried out by Integragen on Illumina NextSeq500 to generate a mean of 150 million reads of 75 base‐pairs per sample. Trimmed reads were then mapped using TopHat2 2.1.0 to the reference genome, and TopHat‐Fusion algorithm was then used to detect RNA fusions. In‐house scripts were used to annotate and translate these gene‐fusions, which were finally validated by blast.

### Reverse transcription polymerase chain reaction for C11orf95‐RELA fusion mRNA

RNA extraction from paraffin‐embedded material and PCR of cDNA was performed for detection of the two most frequently found *RELA‐C11orf* fusion transcripts (Types 1 and II) 19, following protocols previously reported 20, and is explained in detail together with primer sequences in supporting material.

### Comparison of methods and integrated diagnoses

Results of histopathology, p65‐RelA IHC, FISH and DNA methylation‐based classification were analyzed for concordance and used to reach an integrated neuropathological diagnosis for each case. At least two concordant results were required to make a decision. In case of discordance between different methods, further analyses (RT‐PCR and RNA sequencing) were performed if material was available. Imaging was analyzed separately and integrated to the neuropathological diagnosis.

## Results

### Clinico‐pathological features

**Figure 1:**
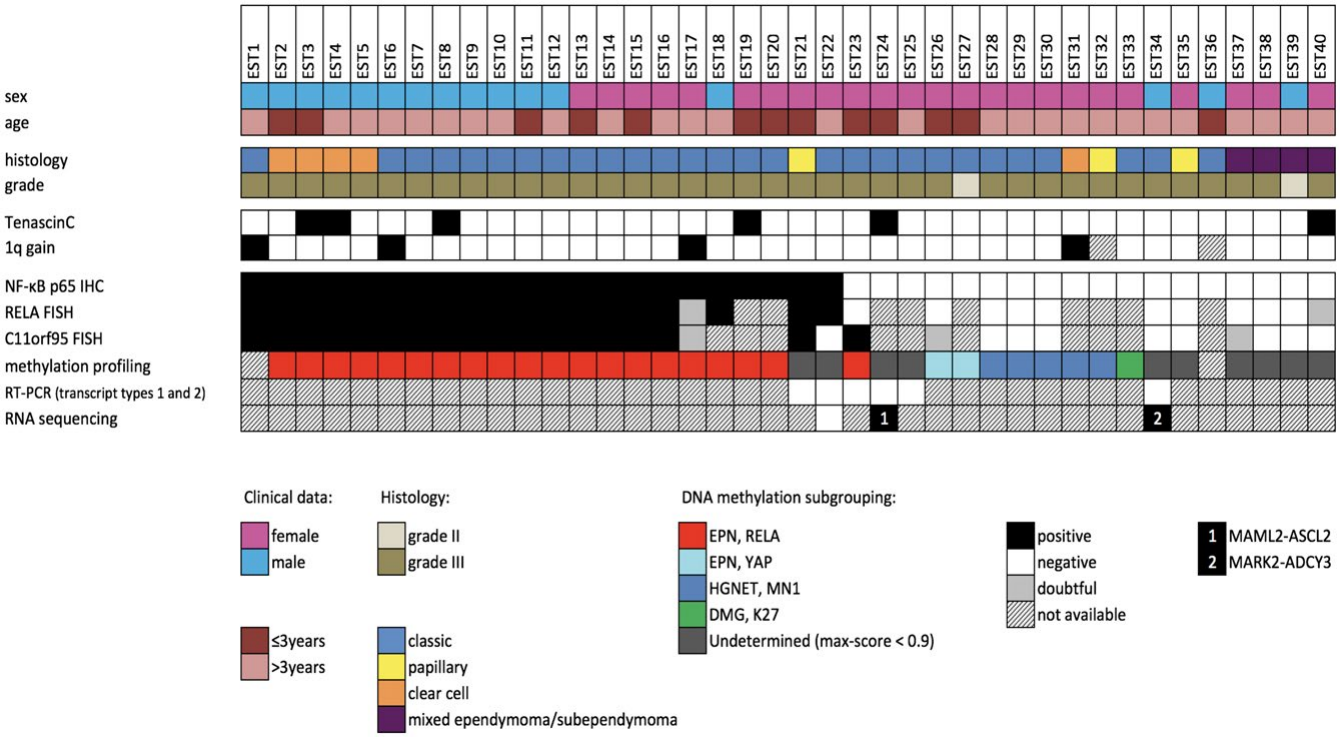
Histopathological features and genetic alterations in 40 pediatric brain tumors diagnosed by histopathological evaluation as supratentorial ependymomas in the past.

Clinical features are summarized in Figure 1 and supplementary Table 1. Our retrospective study cohort comprised 40 patients, including 16 (40%) boys and 24 (60%) girls, with a median age at surgery of 6.5 years (range: 1–17 years). At diagnosis, 13 (32.5%) patients were younger than three years. Gross total resection was achieved for 35 patients. Thirty‐four patients underwent adjuvant treatment by radiotherapy alone (n = 19), chemotherapy alone (n = 6), or radiotherapy associated with chemotherapy (n = 9). According to the WHO 1993, 2000 and 2007 classifications, 38/40 (95%) tumors were assigned to anaplastic WHO grade III ependymomas and 2/40 (5%) were to WHO grade II, and consisted of 28 classic ependymomas, 5 clear‐cell ependymomas, 4 mixed ependymomas/subependymomas and 3 ependymomas with papillary features. Chromosome 1 copy number status demonstrated 1q gain in 4/38 (10.5%) cases.

**Table 1:** Histopathological and molecular profile of ependymomas without evidence of *RELA‐C11orf *or *YAP1 *fusion.

Cases	Age (y)	IHC NF‐κB p65	FISH RELA BA	FISH C11orf95 BA	DNA methylation assay	Chromotripsis chr11	RT‐PCR (transcript type 1 and 2)	RNAsequencing
23	1	−	−	+	EPN, RELA	−	−	ND
24	1	−	Failed	Failed	Undetermined	+	−	MAML2‐ASCL2 t(11;11)(q21;p15.5)
25	8	−	Failed	Failed	Undetermined	−	−	ND
34	10	−	−	−	Undetermined	−	−	MARK2‐ADCY3 t(11;2)(q13.1;p23.3)
35	11	−	−	−	Undetermined	−	ND	ND
36	2	−	Failed	Failed	Failed	NA	ND	ND

Abbreviations: NA = not available; ND = not done.

### Concordance between techniques for the detection of *RELA* ependymomas

**Figure 2:**
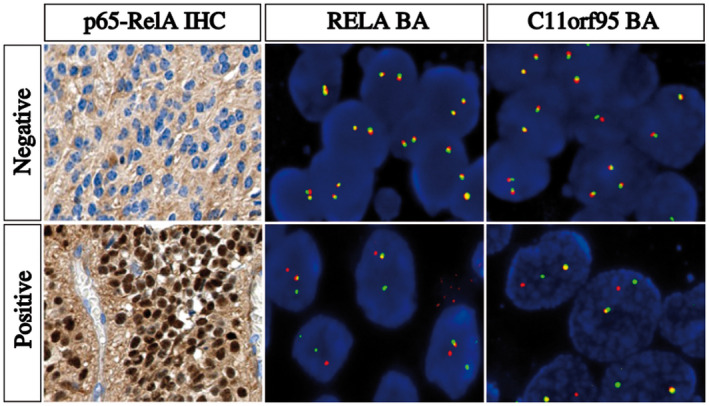
Detection of NFκB pathway activation by IHC and *RELA* and *C11orf95* rearrangements by FISH. IHC and FISH images showing a negative case (top panel) and a positive case (bottom panel). **Left panel**, p65‐RelA IHC images showing an intense nuclear staining in a positive case reflecting an activation of NFκB pathway and a negative case without nuclear staining. **Middle panel**, representative image of a slide hybridized with a *RELA* Break‐Apart FISH probe. In this given example, the images show nuclei harboring a split (red and green signals) and a fused signal in a positive case and two intact fused signals in a negative case. **Right panel**, representative image of a slide hybridized with a *C11orf95* Break‐Apart FISH probe. In this given example, the images show nuclei harboring a split (red and green signals) and a fused signal in a positive case and two intact fused signals in a negative case. IHC, original magnification x40. FISH, Original magnification x1000. IHC, immunohistochemistry; FISH, fluorescence *in situ* hybridization

IHC for p65‐RelA protein was performed for 40 cases, of which 22 (55%) showed nuclear positivity. Twenty of these positive cases displayed strong staining intensity in most neoplastic cell nuclei as shown in Figure 2. Of note, all 115 infratentorial ependymomas tested as controls for comparison were negative for p65‐RelA by IHC.

By FISH analysis, 19 out the 36 (53%) cases tested showed a clear rearrangement of *RELA*, two cases were doubtful, and five cases were non‐informative. Eighteen of the 34 (53%) cases tested with the *C11orf95* break‐apart probe showed a clear rearrangement of *C11orf95*, three cases were doubtful, and the FISH technique failed in four cases. Two cases exhibited only one fusion signal for *RELA* (loss of both 5′ and 3′ signals of one *RELA* locus). One case presented a rearrangement of the *RELA* locus without rearrangement of the *C11orf95* locus. Another case showed a rearrangement of the *C11orf95* locus with a lack of a rearrangement of the *RELA* locus. Positive and negative cases are illustrated in Figure 2.

A DNA methylation profile was obtained for 38 of 40 (95%) cases analyzed, for which a DNA methylation class was determined with certainty (max‐score ≥0.9) for 28 tumors (74%) (Figure 1 and supplementary Table 1). A total of 20 cases were classified as “EPN, RELA” and two as “EPN, YAP.” Five tumors were classified as “HGNET, MN1” tumors and one tumor as a midline diffuse glioma with histone mutation (“DMG, K27”). The DNA methylation class was undetermined for 10 tumors (26%) (Figure 1, supplementary Table 1).

The concordance between IHC for p65‐RelA and *RELA* FISH was 100%. The concordance between p65‐RelA IHC and DNA methylation assay for the detection of the *RELA*‐fused ependymomas was high, with 96.4% agreement (κ = 0.916; CI95[0.754‐1]). Likewise, the concordance between *RELA* FISH and DNA methylation assay for the detection of the *RELA*‐fused ependymomas was high, with an overall agreement of 95.2% (κ = 0.859; CI95[0.592‐1]).

### Integrated diagnosis of ependymal tumors of childhood

#### *RELA*‐fused or *YAP*‐fused ependymomas

Finally, 22 cases were classified as *RELA*‐fused ependymoma (WHO grade III) (Table 2 and supplementary Table 1). In two other cases, occurring in infants (one WHO grade II, case 27 and one WHO grade III, case 26), a *YAP1* rearrangement was detected by PCR and FISH (data not shown). IHC positivity for YAP1 protein was observed in *YAP*‐fused ependymomas as well as in *RELA*‐fused ependymomas and did not allow to distinguish these two subgroups (data not shown). In *RELA*‐fused ependymoma, the DNA methylation‐based classification failed to diagnose with certainty two tumors (#21 and #22) (Table 1). FISH analysis of tumor from patient #21 showed a rearrangement both for *RELA* and *C11orf95* genes in concordance with nuclear p65‐RelA expression. The analysis of the copy number profile of this case revealed chromothripsis of chromosome 11. However, the RT‐PCR for *RELA*‐fusion transcripts type 1 and 2 was negative. A histopathological review was performed and demonstrated ependymal features, namely a pseudopapillary architecture associated with perivascular pseudorosettes. IHC showed strong EMA dot‐like positivity, strong GFAP expression highlighting pseudorosettes and negative OLIG2 staining (data not shown). Taken together, these data led to assume the diagnosis of *RELA*‐fused ependymoma (possibly with a rare alternative *C11orf95*‐*RELA* fusion transcript). Case #22 was a voluminous tumor located both in the supratentorial and infratentorial compartments, and the point of origin could not be determined with certainty by imaging. p65‐RelA nuclear positivity was present and FISH showed a rearrangement in the *RELA* locus. However, no rearrangement was found in the *C11orf95* locus, consistent with the absence of fusion transcripts detected by RT‐PCR. Nevertheless, RNA sequencing failed to detect any other fusion. Copy number alteration analyses revealed a non‐balanced genome including chromosome 11 gain (Table 2 and supplementary Figure 1).

**Table 2:** Integrated diagnosis of supratentorial ependymomas.

Case	Age (y)	Location	Histology		p65RelA IHC	RELA FISH	C11ORF95 FISH	Methylation assay	RT‐PCR	RNAseq	Integrated diagnosis
			Type	Grade							
1	17	NA	Classic	III	1	1	1	Failed	ND	ND	RELA‐fused epend.
2	3	Temporo‐frontal	Clear‐cell	III	1	1	1	EPN, RELA	ND	ND	RELA‐fused epend.
3	1	Parietal	Clear‐cell	III	1	1	1	EPN, RELA	ND	ND	RELA‐fused epend.
4	12	NA	Clear‐cell	III	1	1	1	EPN, RELA	ND	ND	RELA‐fused epend.
5	5	Frontal	Clear‐cell	III	1	1	1	EPN, RELA	ND	ND	RELA‐fused epend.
6	8	Frontal	Classic	III	1	1	1	EPN, RELA	ND	ND	RELA‐fused epend.
7	10	Parietal	Classic	III	1	1	1	EPN, RELA	ND	ND	RELA‐fused epend.
8	7	Frontal	Classic	III	1	1	1	EPN, RELA	ND	ND	RELA‐fused epend.
9	12	Parietal	Classic	III	1	1	1	EPN, RELA	ND	ND	RELA‐fused epend.
10	5	Parietal	Classic	III	1	1	1	EPN, RELA	ND	ND	RELA‐fused epend.
11	2	Occipito‐parietal	Classic	III	1	1	1	EPN, RELA	ND	ND	RELA‐fused epend.
12	5	Fronto‐parietal	Classic	III	1	1	1	EPN, RELA	ND	ND	RELA‐fused epend.
13	1	NA	Classic	III	1	1	1	EPN, RELA	ND	ND	RELA‐fused epend.
14	4	NA	Classic	III	1	1	1	EPN, RELA	ND	ND	RELA‐fused epend.
15	2	Pineal	Classic	III	1	1	1	EPN, RELA	ND	ND	RELA‐fused epend.
16	11	Frontal	Classic	III	1	1	1	EPN, RELA	ND	ND	RELA‐fused epend.
17	7	Parietal	Classic	III	1	Doubtful	Doubtful	EPN, RELA	ND	ND	RELA‐fused epend.
18	9	Frontal	Classic	III	1	1	Failed	EPN, RELA	ND	ND	RELA‐fused epend.
19	2	Temporal	Classic	III	1	ND	ND	EPN, RELA	ND	ND	RELA‐fused epend.
20	3	Temporal	Classic	III	1	Failed	ND	EPN, RELA	ND	ND	RELA‐fused epend.
21	2	NA	Papillary	III	1	1	1	Undetermined	0	ND	RELA‐fused epend.
22	9	Undetermined	Classic	III	1	1	0	Undetermined	0	No fusion	RELA‐fused epend.
23	1	Thalamic‐intrav	Classic	III	0	0	1	EPN, RELA	0	ND	Non RELA/YAP epend.
25	8	Frontal	Classic	III	0	ND	ND	Undetermined	0	ND	Non RELA/YAP epend.
24	1	Tectal	Classic	III	0	Failed	ND	Undetermined	0	MAML2‐ASCL2	Non RELA/YAP epend.
34	10	V3	Classic	III	0	0	0	Undetermined	0	MARK2‐ADCY3	Non RELA/YAP epend.
35	11	Temporo‐parietal	Papillary	III	0	0	0	Undetermined	ND	ND	Non RELA/YAP epend.
36	2	Frontal	Classic	III	0	Failed	Failed	Failed	ND	ND	Non RELA/YAP epend.
27	2	Temporo‐parietal	Classic	II	0	Failed	Failed	EPN, YAP1	ND	ND	YAP‐fused ependymoma
26	0,1	Temporo‐parietal	Classic	III	0	0	Doubtful	EPN, YAP1	ND	ND	YAP‐fused ependymoma
37	10	Parieto‐occipital	Mixed	III	0	0	Doubtful	Undetermined	ND	ND	Epend/subep.mixed tu
38	15	Intraventricular	Mixed	III	0	0	0	Undetermined	ND	ND	Epend/subep.mixed tu
39	4	Parietal	Mixed	II	0	0	0	Undetermined	ND	ND	Epend/subep.mixed tu
40	14	Parieto‐occipital	Mixed	III	0	Doubtful	0	Undetermined	ND	ND	Epend/subep.mixed tu

Abbreviations: epend. = ependymoma; intra = intraventricular, ND = not done; Mixed = ependymal/subependymal mixed tumor; tu = tumor * 450K Max‐Score not in confidence interval.

#### Ependymomas with lack of *RELA* or *YAP* fusion

Six cases (#23, #24, #25, #34, #35, #36) did not harbor *RELA* fusion based on FISH analysis and were negative by IHC for p65‐RelA, suggesting an activation of an alternative pathway in these tumors (Table 2 and supplementary Table 1). DNA methylation profiling classified one case as “EPN, RELA,” which showed a rearrangement of the *C11orf95* locus, suggesting a rearrangement of this gene with another partner than *RELA *(#23). DNA methylation analysis failed to classify with certainty all other cases in this group. In four cases, RT‐PCR was performed on FFPE material and was consistent with IHC and FISH because no specific fusion transcript was found. RNA sequencing analysis identified fusion transcripts, *MAML2‐ASCL2* and *MARK2*‐*ADCY3 *in the two cases for which frozen tissue was available (#24 and #34).

#### Ependymal/subependymal mixed tumors

In four cases (#37, #38, #39, #40), histopathology showed a substantial subependymal component alternating fibrillary or microcystic weakly cellular tissue and more cellular tumor clusters. These could not be annotated to a specific methylation class by methylation profiling with a significant score. However, three of these tumors displayed histopathological signs of malignancy with frequent mitoses, vascular proliferation and necrosis, which are not features of pure subependymoma according to the WHO 2016 guidelines (Supplementary Figure 2). No discrepancy was observed for the detection of *RELA* fusion between IHC/FISH, which were negative, and DNA methylation assay, which failed to classify these tumors with certainty.

#### Other tumors mimicking ependymomas

In this group of tumors, no discrepancy was observed for the detection of *RELA* fusion between IHC/FISH, which were negative, and DNA methylation assay, which classified five of these cases as “HGNET, MN1” (#28, #29, #30, #31, #32), and one as “DMG, K27” (#33).

The five tumors classified as “HGNET, MN1” by DNA methylation profiling were analyzed by FISH using a *MN1* break‐apart probe. A rearrangement of the *MN1* locus was confirmed in four cases (one was not interpretable) (Supplementary Figure 3). Upon histopathological reappraisal, despite perivascular pseudo‐rosettes, negativity for OLIG2 stains and epithelial‐membrane‐antigen dot‐like staining, astroblastic features with prominent perivascular hyalinization, abundant collagen deposition and pseudo‐papillary architecture were present in all five cases.

**Figure 3:**
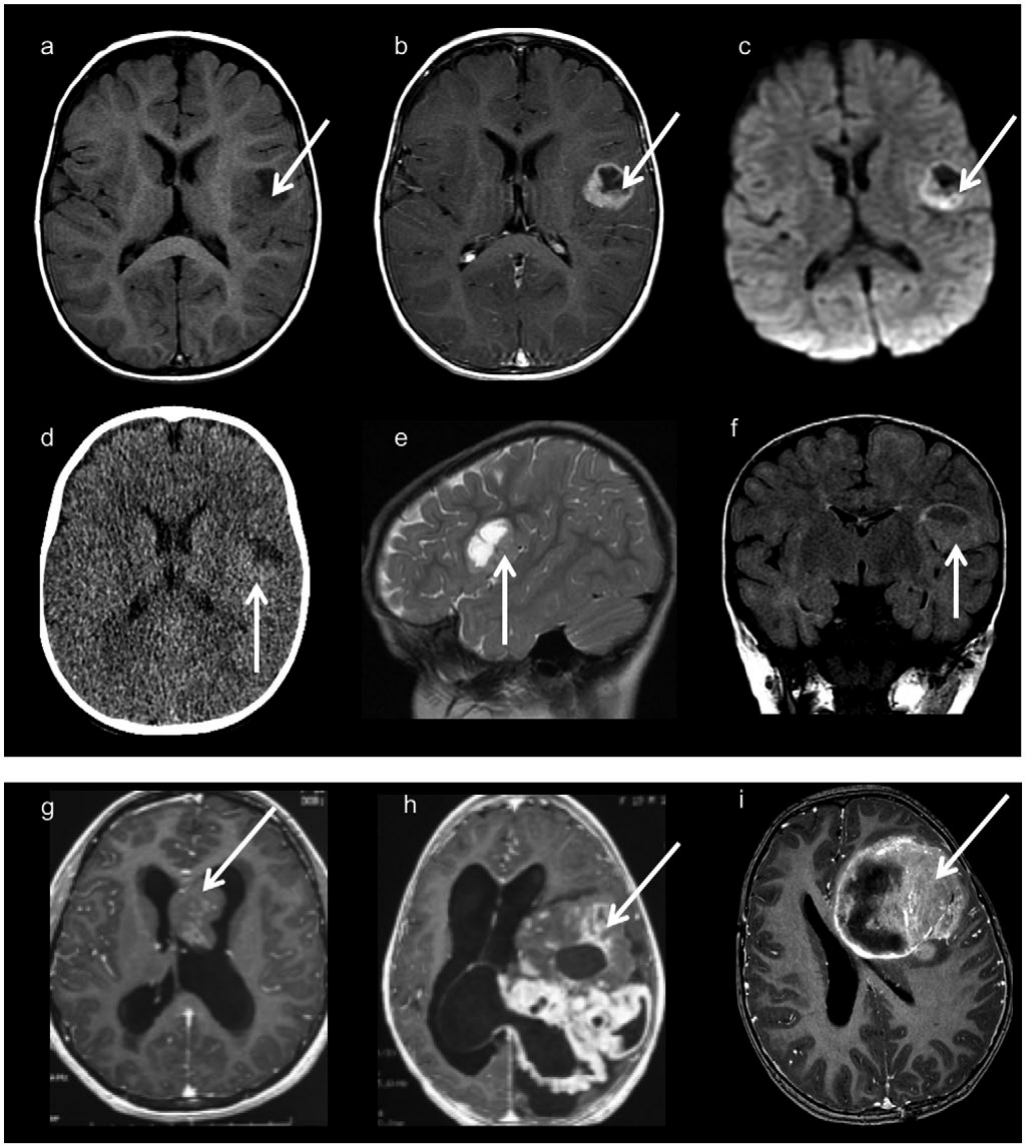
Neuroimaging findings (a–f): Images from one typical supratentorial *RELA*‐fused ependymoma. Axial T1‐weighted images before (a) and after (b) contrast material injection, axial diffusion weighted images (c), CT scan (d), sagittal T2 weighted images (e) and coronal FLAIR images (f). Cortical based, well‐demarcated solid and cystic lesion with a mural nodule and minimal peripheral edema. Contrast injection enhances the nodule and the periphery of the cystic portion. There is diffusion restriction on diffusion‐weighted imaging (c) and a hyper density on the CT scan corresponding to the hyper cellularity. (g): Axial T1 weighted images with contrast injection corresponding to a tumor with mixed ependymal/ subependymal histological features. The intraventricular mass is solid with heterogeneous contrast enhancement. (h): Axial T1‐weighted images with contrast injection from a *YAP*‐fused ependymoma showing a voluminous lesion with prominent solid component with heterogeneous and multinodular appearance. (i): Axial T1‐weighted images with contrast injection from a “HGNET, MN1” tumor, showing a large lesion with a prominent solid portion and necrotic areas.

Upon review, case #33 was a midline tumor in a 10‐year‐old girl operated on in 2003. Histopathological review and additional immunostainings confirmed it to be a histone‐H3 mutated glioma, with histone trimethylation loss and positivity for mutated H3‐K27M protein. However, the striking perivascular arrangement in this case seems rather unusual for a histone‐mutated diffuse midline glioma. Imaging confirmed a midline tumor in the thalamic region.

### Imaging features of *RELA‐fused* ependymoma and other ependymal tumors

Imaging was available for 17/22 *RELA*‐fused ependymomas, of which 13 (76%) showed a similar imaging pattern: a cystic lesion centered in the cerebral cortex with a fairly well‐demarcated small spherical or multilocular mural enhancement, without significant edema (Figure 3a–f). The four *RELA*‐fused ependymomas with different imaging findings were case #7, which had a nodular pattern, #3 showing a very small lesion without a cystic component, #15 which was located in the midline (pineal gland/aqueduct) and # 22, which was located both in the supratentorial and infratentorial compartment, as previously discussed (supplementary Figure 1). From five cases with FLAIR sequences available, four (#1, #6, #9 and #20) had hyperintense intracystic content in this sequence and one showed isointense FLAIR signal in the cystic content (#19). Diffusion restriction was seen in 5/5 patients assessed by diffusion‐weighted imaging and could be explained by tumor hypercellularity (Figure 3c).

In contrast, *YAP*‐fused ependymomas showed a distinct pattern; both tumors were large lesions with a prominent solid component, and multinodular appearance on imaging, distinctive from tumors in the other subgroups (Figure 3h).

The other tumors showed distinct features from *RELA*‐fused ependymomas. Interestingly, three of six tumors reclassified as non‐*RELA*/non‐*YAP* ependymomas were located in the midline (Table 2 and supplementary Table 1). On imaging exams all “HGNET, MN1” were very large lesions with prominent solid portions and necrotic areas; cystic component was not the main tumor feature, in contrast to *RELA*‐fused ependymoma (Figure 3i). Different imaging aspects were seen in tumors with mixed ependymal/subependymal histological features, 2 showing an intraventricular location and solid core (Figure 3g) and two large lesions without significant associated edema and a prominent solid portion, the latter not observed in *RELA*‐fused ependymoma.

### Survival analysis

**Figure 4:**
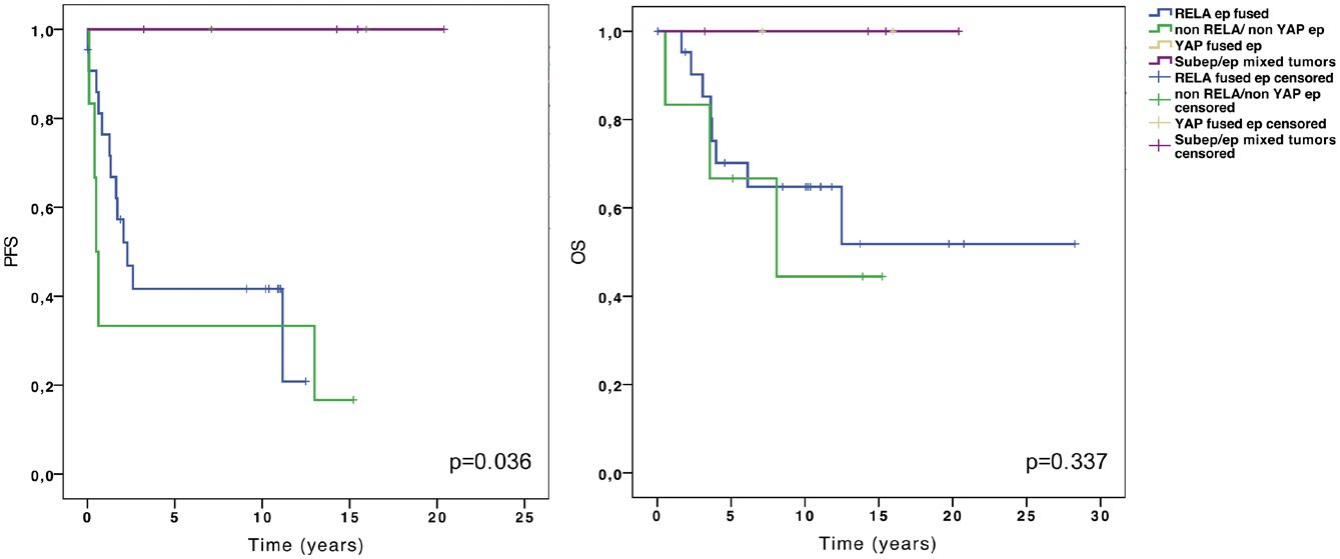
Kaplan–Meier curves of progression‐free survival (PFS) (**left panel**) and overall survival (OS) (**right panel**) stratified by subgroup.

The median follow‐up was 7.2 years (range 0.03–28.3). The 2‐, 5‐ and 10‐year OS and PFS rates were 89.7%, 73.6%, 66.5%, and 59%, 51%, 51%, respectively. No significant association with poor outcome was observed for 1q gain (data not shown). Comparing all tumor subgroups, a significant difference was observed for PFS (*P* = 0.036) but not for OS (*P *= 0.337). *YAP*‐fused ependymomas and ependymal/subependymal mixed tumors were associated with a very good prognosis; these patients exhibited 10‐year OS and PFS rates of 100%. No significant difference was observed between non‐*RELA*/non‐*YAP* ependymomas and *RELA*‐fused ependymomas both for PFS and OS (*P *= 0.532 and *P *= 0.627, respectively) (Figure 4).

## Discussion

### Detection of ependymomas with *RELA* fusion: immunohistochemistry, FISH and DNA methylation classification are complementary methods

Previous studies showed that ependymomas carrying the *C11orf95‐RELA* fusion were characterized by a nuclear accumulation of p65‐RelA, indicating a pathological activation of the NFκB signaling pathway, and reported high correlation with genomic analyses 5, 20. Recently, Gessi *et al* demonstrated the ability of p65‐RelA IHC to predict *RELA* fusion status compared to Sanger sequencing and real‐time polymerase chain reaction 6. However, the specificity of the p65‐RelA IHC positivity was not tested on other ependymomas and histological mimics. In our study, we confirmed the specificity of nuclear p65‐RelA IHC in a series of pediatric supratentorial ependymomas, and further report the absence of p65‐RelA nuclear staining in a large series of control pediatric infratentorial ependymomas, mixed ependymomas/subependymoma, *YAP*‐fused ependymomas as well as in histological mimics including “HGNET, MN1” tumors. Thus, IHC is a simple, sensible and reproducible method that permits to detect a pathological activation of the NFκB pathway, which could be used as a surrogate of the *RELA* fusion in ependymal tumors. Furthermore, the concordance rate of this immunohistochemical marker with FISH was 100% and also very high with DNA‐based methylation classification.

FISH assays are widely used in diagnostic pathology for the detection of genomic rearrangements. However, both *RELA* and *C11orf95* are located on the chromosome 11, approximately 1.9 Mbp apart from each other on the same chromosomal band 11q13. Consequently, the interpretation of FISH break‐apart probe signals for these genes could be tricky. In our series, one case was uncertain for both *RELA* and *C11orf95* rearrangements. FISH using break‐apart probes permits to detect a *C11orf95* and/or *RELA* rearrangement independently of the respective fusion partner and independently of the exact location of the fusion breakpoint. In fact, seven alternative *C11orf95‐RELA* fusion transcripts have been reported, the two most frequent including the exons 1 and 2 of *C11orf95* and the entire open reading frame of *RELA* (except for the first two codons). FISH can detect the fusion regardless of the type of fusion transcript. In contrast, RT‐PCR analysis, which has also shown to be a reliable method 5, may fail in cases exhibiting alternative fusions not specifically covered by the RT‐PCR assay. In our cohort, patient #21 may represent such a case. Results from DNA‐based methylation classification were highly concordant with nuclear p65‐RelA positivity and FISH break‐apart assays. Of note, methylation profiling helped to classify one *RELA*‐fused ependymoma in which FISH yielded doubtful results (#17). On the other hand, DNA methylation classification could not reach scores of sufficient confidence for two *RELA*‐fused ependymomas and misclassified one tumor (#23) as *RELA*‐fused ependymoma, although this case lacked the fusion and showed no evidence for pathological NFkB activation, and therefore by definition did not qualify for this diagnosis.

### Methylation‐based classification can be helpful in the differential diagnosis of ependymal tumors

In this study, classification by methylation profiling provided a powerful tool for the identification of histological mimics of ependymomas, in particular “HGNET, MN1”. Five of such cases could easily be annotated to this methylation subgroup. *MN1* rearrangements could be confirmed in four of these cases, and neuropathological re‐evaluation of these cases showed features of astroblastoma (according to the WHO classification). However, in a significant fraction of the cases in our cohort, the methylation classification failed to securely assign the tumors to a specific entity.

### Imaging data may add another layer of information in the differential diagnosis of *RELA* ependymomas

Imaging may also be helpful in the differential diagnosis of supratentorial brain tumors, especially if a cortical‐based, predominantly cystic lesion is observed, favoring *RELA*‐fused ependymomas 5, 13. For multinodular tumors, particularly in young children, a *YAP*‐fused ependymoma should be ruled out. Subependymal histopathological features should be systematically searched for. Although imaging findings for *RELA*‐fused ependymomas have been reported 5, 13, the specificity and sensitivity of the individual radiological features for the diagnosis of this entity and its differential diagnosis remains to be determined.

### Mixed ependymomas/subependymomas should be diagnostically separated from ependymomas with *RELA* fusions

Subependymomas are rare WHO grade I tumors, often intraventricular, which occur exceptionally in children 9, 14. They are characterized by clusters of isomorphic nuclei with low mitotic activity embedded in a dense fibrillary matrix. These were not included in this study. However, tumors with both subependymoma and classic ependymoma components have been reported 2 and the current WHO classification proposes that such combined tumors should be classified as ependymal/subependymal mixed tumors and graded based on the ependymoma component 9. In our study, all four cases with mixed ependymal and subependymal features did not exhibit NFκB pathway activation or a *C11orf95/RELA* rearrangement. None of these tumors qualified for the histopathologic diagnosis of subependymoma in retrospective evaluation and were diagnosed as supratentorial ependymomas grade II (#39) or III (#37, 38, 40) based on the ependymoma component. Pajtler *et al* mentioned 6 grade II supratentorial ependymomas which clustered in the supratentorial “subependymoma” subgroup (“SUBEPN, ST”) by DNA methylation profiling 17. However, DNA methylation profiling failed to classify our four mixed tumors. Furthermore, by imaging, these tumors showed a prominent solid portion, which was not observed in *RELA*‐fused ependymomas. Interestingly, all patients with these mixed tumors showed an excellent outcome with uneventful PFS and OS. Nevertheless, three of them had histological signs of anaplasia and underwent irradiation and/or chemotherapy, which is not the standard therapy for subependymomas (watch and wait or surgery only). In the study conducted by Pajtler *et al*, all cases classified as “SUBEPN, ST,” including those classified as grade II/III tumors by histopathology, showed uneventful PFS and OS 17. However, all these patients were adults. Due to the rarity of such ependymal/subependymal mixed tumors in children, their molecular pattern and outcome remain unclear 8, 17. Our results argue that the presence of subependymoma areas could indicate the potential for spontaneous differentiation and a favorable outcome. These findings may lead to the hypothesis that these mixed tumors should no longer be graded on the basis of their ependymal component. Focal histopathological features resembling subependymomas may be associated with a better prognosis despite the presence of histopathological signs of anaplasia. This should be further assessed in clinical study cohorts and histopathological grading criteria should be re‐evaluated for such mixed lesions.

### Practical considerations

In practical terms, our results demonstrate that both the detection of nuclear p65‐RelA by immunohistochemistry and break‐apart interphase FISH can be used in clinical diagnostics according to the expertise and skills of the individual pathology laboratory. Both techniques can identify *RELA*‐fused ependymomas, and separate them from non‐*RELA* ependymal tumors or histological mimics. Similarly, FISH for *YAP* fusions could be useful to identify the subgroup of *YAP*‐fused ependymomas, which seems to be associated with a good prognosis in retrospective case descriptions. FISH for *MN1* rearrangement should be performed, if a diagnosis of astroblastoma (WHO classification) or “HGNET, MN1” (methylation subgrouping) is discussed 3, 21. DNA methylation profiling provides a useful layer of information, enabling diagnosis of RELA‐fused ependymomas and helping in the differential diagnosis. However, in this retrospective cohort a significant number of histologically defined supratentorial ependymomas could not be classified by the current DNA methylation profiling algorithms.

To conclude, IHC for nuclear p65‐RelA and FISH using break‐apart probes are highly valuable tools to diagnose *RELA‐*fused ependymomas, which show a worse outcome than ependymomas with *YAP1* fusions and mixed ependymomas/subependymomas.

## Funding

Aurore Besnard was sponsored by BIOMECA consortium (BIOlogical Markers for Ependymomas in Children and Adolescents). JG, DCas, SPi, M‐AD and the molecular profiling were supported by the charity Etoile de Martin and Carrefour through the campaign “Les Boucles du Coeur.”

## Conflict of Interest

The authors declare that they have no conflict of interest except Pascale Varlet (Hoffmann La Roche, Novartis, Boehringer‐Ingelheim) and Torsten Pietsch (Affymetrix/thermos, Roche, Chugai).

## Authors’ Contributions

MP, PV and FA designed the study. MP, KWP, DCas, AT‐E, SPi, M‐AD, MK, TP, PV and FA performed/interpreted the experiments. NB and FA performed the neuroimaging analyses. SPu, CS‐R and JG collected and analyzed clinical data. MP, KWP, SPu, DCas, NB, MK, DCap, FC, SMP, TP, JG, PV and FA performed the analyses of integrated data. All authors provided manuscript review and approval.

## Supporting information


**Figure S1.** Imaging, histological and molecular features of case #22. **a**, Sagittal and coronal T1‐weighted MRI with contrast material injection showing a voluminous tumor developing both in the supratentorial and infratentorial compartment. **b**, p65‐RelA positive IHC with unstained endothelial cell nuclei as internal negative control; original magnification x400. **c**, Representative image of a slide hybridized with a *RELA* Break‐Apart FISH probe showing positive nuclei harboring a split (red and green signals) and two fused signals; original magnification x1000. **d**, Copy number profile from the DNA methylation analysis showing a non‐balanced genome with gain in chromosomes 2, 7, 11, 17, 20 and 21.

**Figure S2.** Representative histopathology of case #37 classified as an ependymal/subependymal mixed tumor. Hematoxylin and eosin‐stained sections exhibited typical subependymoma component, characterized by clusters of small uniform nuclei embedded in a fibrillary matrix (a), with areas showing a higher cellular density with necrosis (b), microvascular proliferation (c) and mitoses (d). Original magnification x200 (a), x400 (b, c, d).

**Figure S3.** Detection of *MN1* rearrangement by FISH. Representative image of a slide hybridized with a *MN1* Break‐Apart FISH probe showing two intact fused signals in a negative case (**a**) and showing nuclei harboring a split (red and green signals) and a fused signal in a positive case (**b**). Original magnification x1000.

**Table S1.** Clinical, histopathological, molecular features and corresponding integrated diagnose. This table includes data from all cases included in the study.

**Methods.** Immunohistochemistry, RNA extraction and RT‐PCR protocols, DNA methylation profiles.
**DNA Methylation Predictions supratentorial tumors.** DNA‐methylation results for all tumors analyzed and their predictions for the diagnosis into different tumor categories.
