# Supplementary figures and images for: Diagnostics of pediatric supratentorial RELA ependymomas: integration of information from histopathology, genetics, DNA methylation and imaging
Source: Brain Pathol. 2018 Nov 28;29(3):325–35. doi: 10.1111/bpa.12664 (PMC7379587; doi:10.1111/bpa.12664)

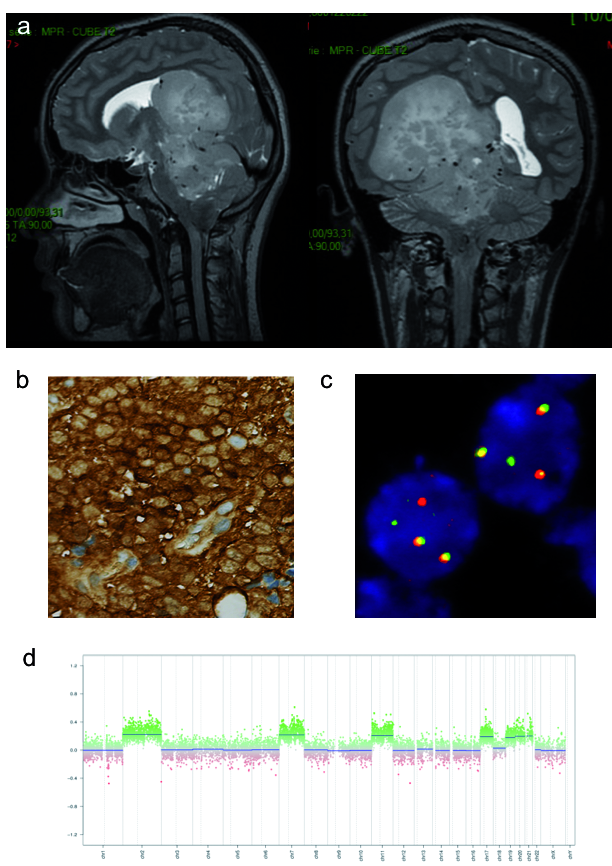

Supplement: Supplementary file 1 — Figure S1. Imaging, histological and molecular features of case #22. a, Sagittal and coronal T1‐weighted MRI with contrast material injection showing a voluminous tumor developing both in the supratentorial and infratentorial compartment. b, p65‐RelA positive IHC with unstained endothelial cell nuclei as internal negative control; original magnification x400. c, Representative image of a slide hybridized with a RELA Break‐Apart FISH probe showing positive nuclei harboring a split (red and green signals) and two fused signals; original magnification x1000. d, Copy number profile from the DNA methylation analysis showing a non‐balanced genome with gain in chromosomes 2, 7, 11, 17, 20 and 21. [file BPA-29-325-s006.tif]

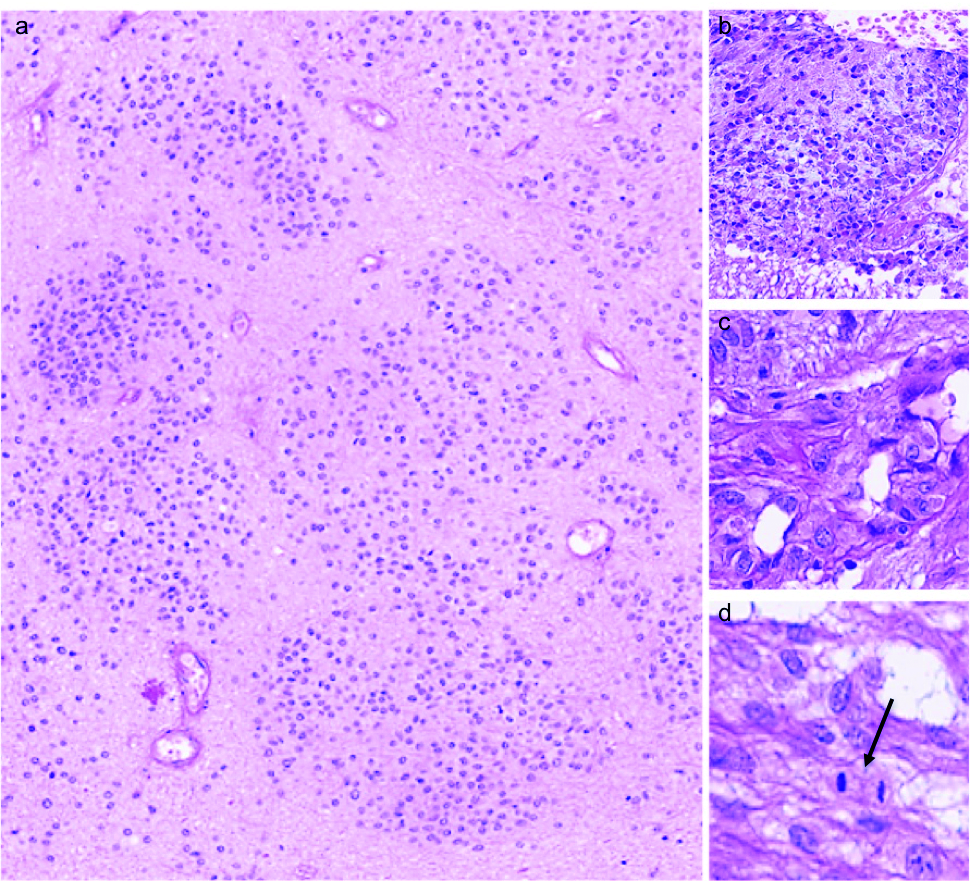

Supplement: Supplementary file 2 — Figure S2. Representative histopathology of case #37 classified as an ependymal/subependymal mixed tumor. Hematoxylin and eosin‐stained sections exhibited typical subependymoma component, characterized by clusters of small uniform nuclei embedded in a fibrillary matrix (a), with areas showing a higher cellular density with necrosis (b), microvascular proliferation (c) and mitoses (d). Original magnification x200 (a), x400 (b, c, d). [file BPA-29-325-s001.tif]

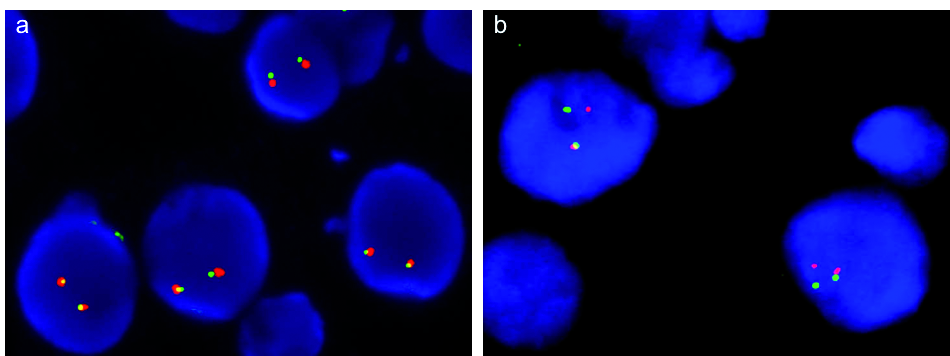

Supplement: Supplementary file 3 — Figure S3. Detection of MN1 rearrangement by FISH. Representative image of a slide hybridized with a MN1 Break‐Apart FISH probe showing two intact fused signals in a negative case (a) and showing nuclei harboring a split (red and green signals) and a fused signal in a positive case (b). Original magnification x1000. [file BPA-29-325-s002.tif]
