# Supplementary material for: Diagnostics of pediatric supratentorial RELA ependymomas: integration of information from histopathology, genetics, DNA methylation and imaging
Source: Brain Pathol. 2018 Nov 28;29(3):325–35. doi: 10.1111/bpa.12664 (PMC7379587; doi:10.1111/bpa.12664)
Supplement: Supplementary file 4 — Table S1. Clinical, histopathological, molecular features and corresponding integrated diagnose. This table includes data from all cases included in the study. [file BPA-29-325-s003.docx]

Supplementary table 1 : clinical, histopathological, molecular features and corresponding integrated diagnosis

| **#** | **Age at diagnosis (y)** | **Location** | **GTR** | **Treatment** | **Histology** | **Grade** | **TNC** | **1q gain** | **p65RelA IHC** | **RELA FISH** | **C11orf95 FISH** | **DNA methylation prediction** | **450K Max-Score** | **RT-PCR** | **RNA-sequencing** | **Integrated diagnosis** |
| --- | --- | --- | --- | --- | --- | --- | --- | --- | --- | --- | --- | --- | --- | --- | --- | --- |
| 1 | 17 | NA | Yes | Rx | Classic | III | 0 | 1 | 1 | 1 | 1 | NA | NA | ND | ND | RELA-fused ependymoma |
| 2 | 3 | Temporo-frontal | Yes | Rx+C | Clear cells | III | 0 | 0 | 1 | 1 | 1 | EPN, RELA | 0.999 | ND | ND | RELA-fused ependymoma |
| 3 | 1 | Parietal | Yes | C | Clear cells | III | 1 | 0 | 1 | 1 | 1 | EPN, RELA | 0.999 | ND | ND | RELA-fused ependymoma |
| 4 | 12 | NA | Yes | Rx | Clear cells | III | 1 | 0 | 1 | 1 | 1 | EPN, RELA | 0.989 | ND | ND | RELA-fused ependymoma |
| 5 | 5 | Frontal | Yes | Rx | Clear cells | III | 0 | 0 | 1 | 1 | 1 | EPN, RELA | 0.999 | ND | ND | RELA-fused ependymoma |
| 6 | 8 | Frontal | Yes | Rx | Classic | III | 0 | 1 | 1 | 1 | 1 | EPN, RELA | 0.999 | ND | ND | RELA-fused ependymoma |
| 7 | 10 | Parietal | Yes | Rx | Classic | III | 0 | 0 | 1 | 1 | 1 | EPN, RELA | 0.999 | ND | ND | RELA-fused ependymoma |
| 8 | 7 | Frontal | No | Rx | Classic | III | 1 | 0 | 1 | 1 | 1 | EPN, RELA | 0.999 | ND | ND | RELA-fused ependymoma |
| 9 | 12 | Parietal | Yes | Rx | Classic | III | 0 | 0 | 1 | 1 | 1 | EPN, RELA | 0.991 | ND | ND | RELA-fused ependymoma |
| 10 | 5 | Parietal | Yes | C | Classic | III | 0 | 0 | 1 | 1 | 1 | EPN, RELA | 0.999 | ND | ND | RELA-fused ependymoma |
| 11 | 2 | Occipito-parietal | Yes | Rx+C | Classic | III | 0 | 0 | 1 | 1 | 1 | EPN, RELA | 0.999 | ND | ND | RELA-fused ependymoma |
| 12 | 5 | Fronto-parietal | Yes | Rx | Classic | III | 0 | 0 | 1 | 1 | 1 | EPN, RELA | 0.999 | ND | ND | RELA-fused ependymoma |
| 13 | 1 | NA | No | Rx+C | Classic | III | 0 | 0 | 1 | 1 | 1 | EPN, RELA | 0.999 | ND | ND | RELA-fused ependymoma |
| 14 | 4 | NA | No | None | Classic | III | 0 | 0 | 1 | 1 | 1 | EPN, RELA | 0.999 | ND | ND | RELA-fused ependymoma |
| 15 | 2 | Pineal | Yes | C | Classic | III | 0 | 0 | 1 | 1 | 1 | EPN, RELA | 0.999 | ND | ND | RELA-fused ependymoma |
| 16 | 11 | Frontal | Yes | Rx | Classic | III | 0 | 0 | 1 | 1 | 1 | EPN, RELA | 0.999 | ND | ND | RELA-fused ependymoma |
| 17 | 7 | Parietal | No | Rx | Classic | III | 0 | 1 | 1 | Doubtful | Doubtful | EPN, RELA | 0.999 | ND | ND | RELA-fused ependymoma |
| 18 | 9 | Frontal | Yes | Rx | Classic | III | 0 | 0 | 1 | 1 | Failed | EPN, RELA | 0.999 | ND | ND | RELA-fused ependymoma |
| 19 | 2 | Temporal | Yes | Rx+C | Classic | III | 1 | 0 | 1 | ND | ND | EPN, RELA | 0.999 | ND | ND | RELA-fused ependymoma |
| 20 | 3 | Temporal | Yes | C | Classic | III | 0 | 0 | 1 | Failed | ND | EPN, RELA | 0.999 | ND | ND | RELA-fused ependymoma |
| 21 | 2 | NA | Yes | None | Papillary | III | 0 | 0 | 1 | 1 | 1 | Undetermined | 0.293 | 0 | ND | RELA-fused ependymoma |
| 22 | 9 | undetermined | Yes | Rx+C | Classic | III | 0 | 0 | 1 | 1 | 0 | Undetermined | 0.297 | 0 | No fusion | RELA-fused ependymoma |
| 23 | 1 | Thalamic-intraV | No | C | Classic | III | 0 | 0 | 0 | 0 | 1 | EPN, RELA | 0,977 | 0 | ND | Non RELA/YAP ependymoma |
| 24 | 1 | Tectal | Yes | Rx+C | Classic | III | 1 | 0 | 0 | Failed | ND | Undetermined | 0.812 | 0 | MAML2-ASCL2 | Non RELA/YAP ependymoma |
| 25 | 8 | Frontal | Yes | C | Classic | III | 0 | 0 | 0 | ND | ND | Undetermined | 0.397 | 0 | ND | Non RELA/YAP ependymoma |
| 26 | 0,1 | Temporo-parietal | Yes | None | Classic | III | 0 | 0 | 0 | 0 | Doubtful | EPN, YAP | 0.999 | ND | ND | YAP-fused ependymoma |
| 27 | 2 | Temporo-parietal | Yes | None | Classic | II | 0 | 0 | 0 | Failed | Failed | EPN, YAP | 0.999 | ND | ND | YAP-fused ependymoma |
| 28 | 7 | V3 | Yes | Rx | Classic | III | 0 | 0 | 0 | 0 | 0 | HGNET, MN1 | 0.991 | ND | ND | HGNET |
| 29 | 4 | Frontal | Yes | Rx | Classic | III | 0 | 0 | 0 | 0 | 0 | HGNET, MN1 | 0.998 | ND | ND | HGNET |
| 30 | 11 | Frontal | Yes | Rx | Classic | III | 0 | 0 | 0 | 0 | 0 | HGNET, MN1 | 0.997 | ND | ND | HGNET |
| 31 | 6 | Temporo-parietal | Yes | Rx | Clear cells | III | 0 | 1 | 0 | ND | ND | HGNET, MN1 | 0.999 | ND | ND | HGNET |
| 32 | 17 | NA | Yes | Rx+C | Papillary | III | 0 | Failed | 0 | ND | ND | HGNET, MN1 | 0.977 | ND | ND | HGNET |
| 33 | 10 | Thalamic | No | Rx+C | Classic | III | 0 | 0 | 0 | Failed | ND | DMG, K27 | 0.999 | ND | ND | H3.3 K27M glioma |
| 34 | 10 | V3 | Yes | Rx | Classic | III | 0 | 0 | 0 | 0 | 0 | Undetermined | 0.871 | 0 | MARK2-ADCY3 | Non RELA/YAP ependymoma |
| 35 | 11 | Parieto-temporal | Yes | Rx+C | Papillary | III | 0 | 0 | 0 | 0 | 0 | Undetermined | 0.104 | ND | ND | Non RELA/YAP ependymoma |
| 36 | 2 | Frontal | Yes | None | Classic | III | 0 | Failed | 0 | ND | Failed | NA | NA | ND | ND | Non RELA/YAP ependymoma |
| 37 | 10 | Parieto-occipital | Yes | None | Mixed | III | 0 | 0 | 0 | 0 | Doubtful | Undetermined | 0.114 | ND | ND | Epend/subep. mixed tumour |
| 38 | 15 | Intraventricular | Yes | Rx | Mixed | III | 0 | 0 | 0 | 0 | 0 | Undetermined | 0.145 | ND | ND | Epend/subep. mixed tumour |
| 39 | 4 | Parietal | Yes | Rx | Mixed | II | 0 | 0 | 0 | 0 | 0 | Undetermined | 0.369 | ND | ND | Epend/subep. mixed tumour |
| 40 | 14 | Parieto-occipital | Yes | Rx | Mixed | II | 1 | 0 | 0 | Doubtful | 0 | Undetermined | 0.203 | ND | ND | Epend/subep. mixed tumor |
| C: chemotherapy; Epend.: ependymoma; ND: not done; NA: not available; ND: not done; Rx: radiotherapy; Epend/subep. mixed tumour: ependymal/subependymal mixed tumour; *not in confidence interval | | | | | | | | | | | | | | | | |
