# Supplementary material for: Diagnostics of pediatric supratentorial RELA ependymomas: integration of information from histopathology, genetics, DNA methylation and imaging
Source: Brain Pathol. 2018 Nov 28;29(3):325–35. doi: 10.1111/bpa.12664 (PMC7379587; doi:10.1111/bpa.12664)
Supplement: Supplementary file 6 — Methods. Immunohistochemistry, RNA extraction and RT‐PCR protocols, DNA methylation profiles. DNA Methylation Predictions supratentorial tumors. DNA‐methylation results for all tumors analyzed and their predictions for the diagnosis into different tumor categories. [file BPA-29-325-s005.docx]

Supplementary material- Methods

Immunohistochemistry

Histological sections were deparaffinised and processed with a Ventana autostainer (BenchMark XT, Ventana Medical Systems or Discovery XT, Ventana Medical Systems, Oro Valley, U.S.A.) according to a standard protocol. Antigen retrieval was performed for 30 minutes (CC1, pH8 ,Ventana Medical Systems), followed by incubation with anti-p65-RelA monoclonal antibody (D14E12, 1/3000, Cell signaling, Danvers, U.S.A.) for 24 minutes. Immunostaining for Yap1 protein (#sc-101199, Santa Cruz, Dallas, U.S.A.; diluted at 1/50) was also performed as well as for BCOR (C-10; diluted at 1/100, Santa Cruz). IHC for Tenascin C, H3K27M and H3K27me3 have been performed as previously described [3,12]. p65-RelA IHC was independently analysed by two senior pathologists (FA and PV) blinded for the FISH and DNA methylation results. The cases were considered p65-RelA positive if a nuclear staining was observed in at least 15% of tumour cells. Cytoplasmic staining was not taken into account. 115 infratentorial paediatric ependymoma samples included in 3 tissue microarray (TMA) blocks were used as negative controls for p65-RelA immunohistochemistry.

RNA extraction and RT-PCR

Total RNA of ependymoma cases was extracted from FFPE tissue using the AllPrep® DNA/RNA FFPE Kit from Qiagen (Hilden, Germany) according to the manufacturers' instructions. 250ng of total RNA (as measured by 260nm extinction) were then reverse-transcribed using the TaKaRa Prime Script RT reagent Kit (Perfect Real Time; Takara Bio Inc., Saint-Germain-en-Laye, France) and random primers.

PCR of cDNA was performed with primers located in exon 2 of *C11orf95* (5' aggaagtcatcagcaacagc 3'), and in exon 3 of *RELA* (5' tcttggtggtatctgtgctc 3') Primers located in exon 3 of *C11orf95* (5' cctgcacctggacgacat 3') and in exon 3 of *RELA* (5' gctgctcaatgatctccaca 3') were also used to detect an alternative transcript (type II) [7]. PCR was performed under following conditions: annealing temperature, 65°C; elongation, 72°C for 30 sec; 50 cycles. The generated PCR fragments were analysed on a 2 % agarose gel. PCR products were visualised and documented on a Geldoc 1000 system (Biorad, Munich, Germany). PCR products were purified using a PCR purification kit (Qiagen). Direct Sanger sequencing reactions were performed in duplicate (forward and reverse).

DNA methylation analysis

DNA methylation profiling was performed at the DKFZ Genomics and Proteomics Core Facility (Heidelberg, Germany) utilising the Illumina HumanMethylation450 BeadChip array (450k array) (Illumina, San Diego, U.S.A.) according to the manufacturer’s instructions. The resulting data were processed and samples were classified into molecular subgroups as previously described (3).
